# Safety and Feasibility of a Novel Split Design Left Atrial Appendage Occluder: From Preclinical Investigations to First‐in‐Human Application

**DOI:** 10.1155/cdr/1340277

**Published:** 2026-04-27

**Authors:** Mingfei Li, Dawei Lin, Lei Zhang, Yuan Zhang, Wenzhi Pan, Xiaochun Zhang, Daxin Zhou, Junbo Ge

**Affiliations:** ^1^ Department of Cardiology, Zhongshan Hospital, Fudan University, Shanghai Institute of Cardiovascular Diseases, Shanghai, China, zs-hospital.sh.cn; ^2^ State Key Laboratory of Cardiovascular Diseases, Zhongshan Hospital, Fudan University, Shanghai, China, fudan.edu.cn; ^3^ NHC Key Laboratory of Ischemic Heart Diseases, Shanghai, China; ^4^ Key Laboratory of Viral Heart Diseases, Chinese Academy of Medical Sciences, Shanghai, China, cacms.ac.cn; ^5^ National Clinical Research Center for Interventional Medicine, Shanghai, China

**Keywords:** animal experiments, atrial fibrillation, left atrial appendage occlusion, the split occluder

## Abstract

**Objective:**

This study was designed to first evaluate the histological compatibility of the split occluder in preclinical animal models, followed by clinical validation of its procedural feasibility and occlusion effectiveness in a first‐in‐human trial cohort.

**Methods:**

Eight healthy labrador retrievers were selected for this study. A split‐type occluder was introduced to occlude their left atrial appendage. The experimental animals were euthanized at 1, 3, and 6 months, respectively. Prior to being euthanized, transesophageal echocardiogram (TEE) and pulmonary vein computed tomography venography (CTV) were conducted on the subjects. A gross anatomical examination was then conducted on each animal post euthanasia. Additionally, data from 12 patients who underwent LAAO procedures using this device were included in the study. Each patient was monitored over a 3‐month follow‐up period.

**Results:**

The operations to insert split left atrial appendage occluders into each of the eight test dogs were successful. Subsequent TEE and pulmonary vein CTV revealed that occluder surfaces exhibited no thrombus formation and that the occluders exhibited no significant residual flow. The gross anatomical examination indicated satisfactory endothelialization. The occluders were successfully implanted in all 12 patients, with no complications reported in the occlusions during the subsequent 3‐month follow‐up period.

**Conclusions:**

In both animal and human studies, the split occluder demonstrated a significantly high immediate success rate in the occlusion of the left atrial appendage. This was accompanied by a low occurrence of residual shunts and device‐related complications, indicating its reliability and efficacy.

## 1. Introduction

Atrial fibrillation (AF), a common arrhythmia, increases in incidence with age [1]. As of 2019, AF and atrial flutter were estimated to affect approximately 59.7 million individuals globally. Specifically, one in three people aged 55 and above are affected, with prevalence standing at 0.60% for men and 0.37% for women [2] when factoring in adjusted age rates. Notably, for those suffering from AF, thromboembolic complications are the leading cause of death and disability, of which ischemic stroke is the most common form.

For patients with nonvalvular atrial fibrillation (NVAF), left atrial appendage occlusion (LAAO) is now emerging as an effective means to lower the incidence of ischemic stroke. It is an alternative treatment for patients at high risk of contraindications to anticoagulation and exhibits an efficacy comparable with that of warfarin [3, 4]. Currently, LAAO available for sale on foreign markets mainly comprise the Watchman and Amulet occluders, which have been successfully implanted in tens of thousands of clinical operations [5, 6]. The LAmbre occluder from Lifetech (Shenzhen) Co. Ltd. and the LACbes occluder from Push (Shanghai) Medical Device Co. Ltd. are available on the Chinese market and have also been utilized in clinical practice [7, 8].

However, challenges arise when existing devices do not match the varied sizes of left atrial appendage (LAA) morphology, which can lead to occlusion failure. A novel split LAA occluder presents a promising solution. Its design allows for intraoperative customization, enabling the selection of an occluder that best conforms to the individual patient′s LAA anatomy. The device we have developed is adaptable to a wide range of LAA forms and structures. This study consists of preliminary animal experiments and human trials that explore the feasibility and effectiveness of this split LAA occluder when inserted into a LAAO.

## 2. Methods

### 2.1. Device Design

Our nickel–titanium alloy wire–woven split occluder comprises unique design of a covering disc and a fixed disc (Figure 1). The fixed disc and covering disc can be deployed separately. The fixed disc functions independently as a plug‐type occluder when used in isolation, or can be combined with the covering disc to form a covered double‐disc occluder (Figure 1a,b). Figure 1c,d shows the gross appearance of the occluder. When employing the plug‐type configuration without the covering disc, the modular design allows flexible dimensional matching between the fixed disc (primary anchoring component) and covering disc parameters. This adaptability enables customized size combinations according to specific anatomical requirements in either operational mode. As a cover‐type double‐disc occluder, the fixed disc and covering disc are interconnected via a threaded screw–nut interface (Figure 1b), typically requiring five to six clockwise rotations to achieve secure fixation. During device detachment from the delivery system (which necessitates counterclockwise rotation), the counterclockwise detachment force may potentially affect the connection integrity between the discs. Furthermore, postimplantation cardiac motion could induce relative displacement between the discs during cardiac cycles, thereby increasing the risk of connection loosening at the disc interface. To address these challenges, the modular LAA occluder incorporates an innovative locking mechanism at the disc junction. As shown in Figure 1b, the nut component of the fixed disc features precision‐engineered grooves, whereas the screw base of the covering disc is equipped with right‐angled triangular protrusions. When the discs are fully tightened through clockwise rotation, these geometric protrusions engage with the corresponding grooves, creating a positive mechanical lock. This interlocking design ensures: (1) prevention of unintended counterclockwise rotation during device detachment; (2) elimination of relative motion between discs during cardiac contraction; and (3) maintenance of long‐term connection stability through geometric interference, thereby guaranteeing reliable coupling of the dual‐disc system throughout the device lifecycle. The fixed and covering discs provide a snug fit via their securely connected screws and nuts. The split LAA occluder support mesh is entirely constructed from a nickel–titanium alloy wire, offering both durability and flexibility. The occluder utilized in clinical trials is available in 13 specifications, categorized by the diameter of the fixed disc, ranging from 14 to 38 mm, in 2 mm increments. The diameter of the covering disc ranges from 20 to 44 mm, again in 2 mm increments. Additionally, customization is possible via the flexibility in assembling different fixed disc models. For this experiment, the chosen specifications included fixed disc sizes of 22 and 26 mm, whereas dual disc sizes ranged from 16 to 28 mm and 20 to 30 mm.

Figure 1The schematic diagrams and actual pictures of the split occluder. (a) A schematic diagram of the fixed and covering discs of the device. (b) A schematic diagram of the device when the fixed and covering discs are connected. (c) When the fixed and the covering discs of the device are separated. (d) When the fixed and the covering discs of the device are combined.

**Figure:**
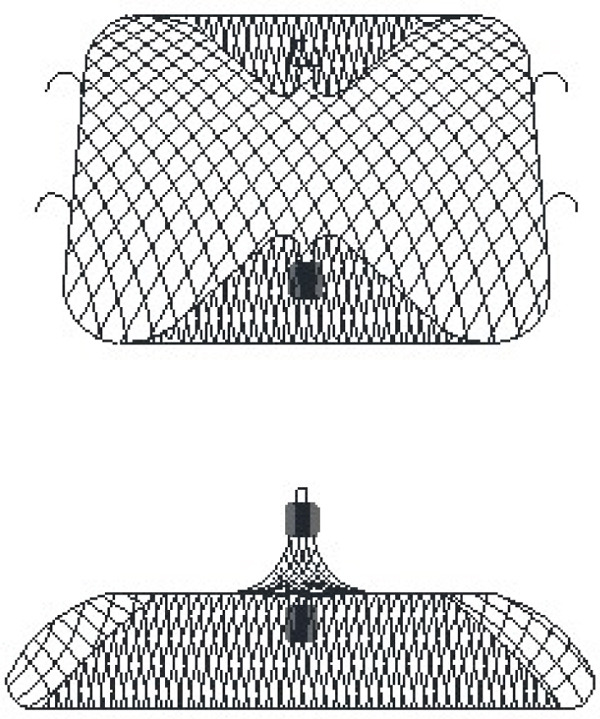
(a)

**Figure:**
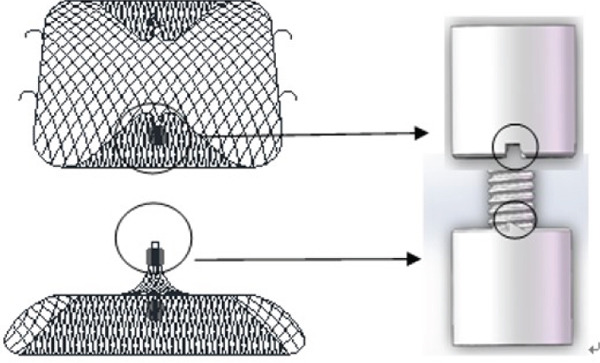
(b)

**Figure:**
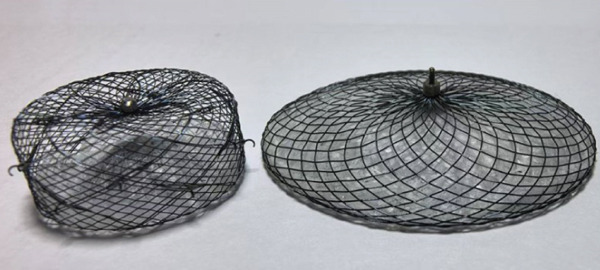
(c)

**Figure:**
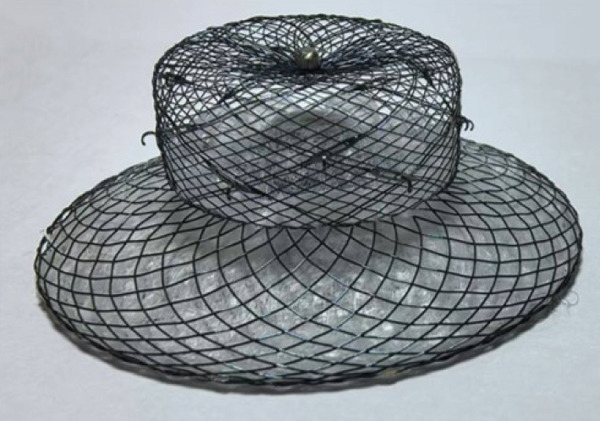
(d)

### 2.2. Animal Experiment

#### 2.2.1. Animal Model

Between May and October 2023, eight labrador dogs, five males and three females, weighing (30.2 ± 2.3) kg, were entered into this study. These dogs were provided by Zhejiang Hongfeng Biotechnology Co. Ltd. and were cared for in an appropriate, clean environment. All experimental procedures were approved by the Fudan University Experimental Animal Care and Use Committee.

#### 2.2.2. Animal Procedure

**Figure 2:**
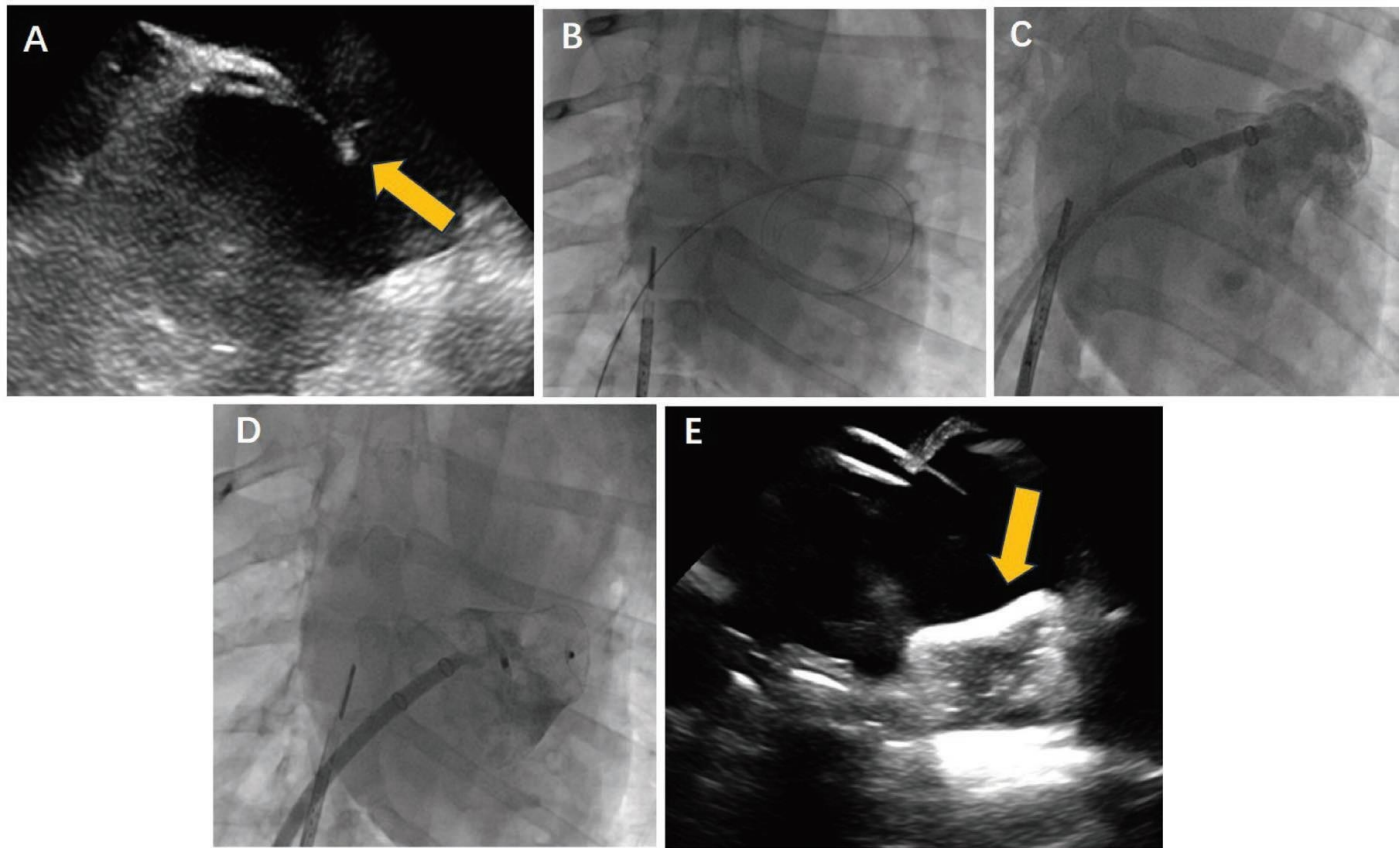
ICE and fluoroscopy‐guided LAAO with the split occluder in animal. (A) Atrial septal puncture under ICE guidance (arrow points to the atrial septal puncture needle). (B) The wire was successfully inserted through the atrial septum. (C) Left atrial appendage angiography was performed. (D) The occluder was deployed under fluoroscopy. (E) ICE showed that the occluder was in a stable position with no significant residual shunt (arrow points to the occluder).

The dogs were fasted for a period of 12 h prior to the experiment start. Isoflurane (0.05 mL/kg) and atropine (0.02 mg/kg) were administered via intramuscular injection to induce anesthesia. The dogs were then secured to a digital subtraction angiography (DSA) bed. The site surrounding the bilateral inguinal areas was cleared of fur and thoroughly disinfected, following which sterile drapes were applied. Subsequently, zolazepam (dosed at 0.5–0.6 mg/100 g) was administered to maintain anesthesia throughout the procedure by puncturing the superficial veins of their limbs. Various vital signs were continuously monitored, including respiration rate, heart rate, body temperature, and blood oxygen saturation, as well as an electrocardiogram. Small‐gauge needles were then inserted into the superficial arteries of the limbs to facilitate the continuous monitoring of invasive arterial pressure. A direct approach was taken to cut open the femoral vein on one side and insert a 10F sheath for the delivery of an intracardiac echocardiography (ICE) catheter. This allowed for precise observation of the left atrium, as well as LAA structures within the right atrium. Proceeding with a more detailed procedure, the femoral vein on one side was punctured using a 6F sheath under direct vision. A guide wire was then inserted, whereupon the 6F sheath was withdrawn and replaced with an 8F atrial septal puncture sheath. An atrial septal puncture was executed (Figure 2a) guided by an ICE catheter. A steel wire with two and a half turns to feed the inner sheath into the left atrium (Figure 2b). After feeding the Swartz sheath into the left atrium, the steel wire was kept in place while the atrial septal puncture sheath was removed. Once sure that the atrial septal puncture needle had successfully entered the left atrium, heparin (100–150 IU/kg) was administered intravenously to activate the dog′s systemic blood. Following this, 2 mL of arterial blood was collected 10 min later for blood gas and activated clotting time (ACT) testing. The target ACT value was maintained for between 250 and 350 s. Next, a 12F LAA delivery sheath was inserted along the left atrium wire with the inner sheath withdrawn. Subsequently, a 6F pigtail catheter was fed into the LAA along the wire. After extracting the wire, a LAA angiography was performed at specific projection angles (RAO25° and CAU15°) to assess the morphology of the LAA, as well as its maximum inner and outer diameters (Figure 2c). The split occluder was then inserted via the delivery sheath and guided to the LAA using DSA or ICE for auxiliary positioning. Once the occluder was properly positioned, a high‐pressure injection separated the fixed and covering discs for angiography to observe the implantation. A push–pull test was conducted to confirm the occluder′s stability. Re‐angiography was performed to ensure that the occluder was in an optimal position, and there were no residual shunts remaining. The occluder was then released once everything was satisfactory (Figure 2d,e). The sheath was withdrawn postprocedure, and vital signs were closely monitored. The puncture site on the right femoral vein was then sutured and dressed, whereas the femoral artery was punctured and ligated to control bleeding. Antibiotics were then administered on the day of the procedure and for five subsequent days to minimize the risk of infection. Additionally, low‐molecular weight heparin (1000 U/12 h) was administered subcutaneously 3 days postprocedure. All dogs in the experiment were prescribed 50‐mg aspirin enteric‐coated tablets and 37.5‐mg clopidogrel bisulfate tablets daily until the end of the trial.

#### 2.2.3. Evaluation Protocol

The device implantation was deemed a success when the occluder deployed from the delivery system and maintained a stable position within the LAA, and an ICE examination demonstrated that there was no residual flow around the occlusion and no adverse effects were evident on adjacent anatomical structures. Adverse events encompassed death, pericardial tamponade, device migration, and occluder‐related complications such as thrombosis or embolization during either the procedure or follow‐up. Two dogs were euthanized 1 month postoperation, and three dogs were euthanized at both the subsequent 3‐ and 6‐month milestones. TEE and pulmonary venous computed tomography venography (CTV) were conducted to assess the stability of the occluder, the occurrence of mural thrombus, and any residual shunts on each animal immediately prior to their euthanasia.

### 2.3. First‐in‐Human Study

#### 2.3.1. Study Design and Patients

For this prospective, single‐center, exploratory clinical study, 12 patients with NVAF were recruited from Fudan University Affiliated Zhongshan Hospital between March and September 2024. The study was approved by the Ethics Committee of Zhongshan Hospital, Fudan University (NO.2023‐120R2), and all participants submitted written informed consent. These patients were either unsuited or unwilling to take long‐term oral anticoagulant therapy. Furthermore, some patients remained at risk of stroke or thromboembolism even after receiving this treatment, with CHA2DS2‐VASc scores of 2 or higher (3 or higher for females). Following TEE and fluoroscopy guidance, they underwent the procedure, with the split occluder employed to treat a LAAO. A TEE examination was then conducted 90 days postprocedure. Patients initially received an anticoagulation course of 15‐mg rivaroxaban tablets for 2 months, and then a course of 100‐mg aspirin and 75‐mg clopidogrel for 4 months. Reported adverse events for this approach encompassed death, pericardial tamponade, device migration, device‐related thrombosis, embolization, infection, and vascular complications. Acute procedure success was defined as the successful implantation of the device with no or minimal subsequent peridevice leak (PDL). The primary efficacy endpoint consisted of a composite measure comprised of acute procedure success with no or minimal PDL at 90 days. Safety was defined as the absence of adverse events during the follow‐up period. The stability of the occluder, the presence of mural thrombus, and PDL were assessed using TEE and pulmonary venous CTV.

#### 2.3.2. Procedure

The device was implanted for a LAAO with the aid of TEE and fluoroscopic guidance. The choice of occluder type and size was determined by the morphology and dimensions of the patients′ LAAs. The decision to increase the size of a device by 4–6 mm was dependent on the largest diameter of the LAA ostium, measured at specific angles during intraprocedural angiography and TEE. This measurement was crucial for selecting the appropriate device size. In cases where the available depth was limited, a smaller sized device might be considered following a comprehensive LAA evaluation. Once selected, the device was deployed under fluoroscopic guidance and was evaluated for proper positioning and functionality prior to final ejection. This clinical surveillance was essential to ensure that the LAA occlusion device met the criteria of PAST (Proper Position–Absolute Anchor–Separate Seal–Typical Tire).

### 2.4. Statistical Analysis

Continuous data were presented in the format of the mean ± standard deviation and were analyzed using either the Student′s *t*‐test or the Mann–Whitney *U* test, as appropriate. Quantitative data were, depending on the data characteristics, described in terms of frequencies and percentages, with comparisons conducted using the chi‐square test or Fisher′s exact test. Notably, all *p* values were two‐sided, with a value of *p* < 0.05 being considered statistically significant. This statistical analysis was performed with the SPSS 26.0 program.

## 3. Results

### 3.1. Results of Animal Experiments

The LAA angiography of the eight dogs revealed that all possessed single‐leaf LAAs (comprising six chicken wing types and two windsock types). The measured dimensions included an outer opening with an inner diameter of (20.4 ± 1.2) mm and an inner opening with an inner diameter of (13.2 ± 1.1) mm. Notably, the occluders successfully achieved a complete occlusion of the LAAs in all canine test subjects. Specifically, four dogs were fitted with a fixed disc of either 22 or 26 mm, whereas the remaining four dogs were fitted with a combination of a fixed disc and a covering disc with occluders ranging from 20 to 28 mm and 20 to 30 mm. The immediate occlusion success rate for this batch of procedures was 100%. Seven of the dogs exhibited no evident residual shunt, whereas one displayed a minor shunt. During the subsequent 1–6‐month follow‐up periods, all eight dogs demonstrated normal behavior, development, and activity levels, and did not display any signs of hemiplegia, drooling, or other symptoms indicative of stroke.

### 3.2. The Acute and Follow‐Up Results

**Figure 3:**
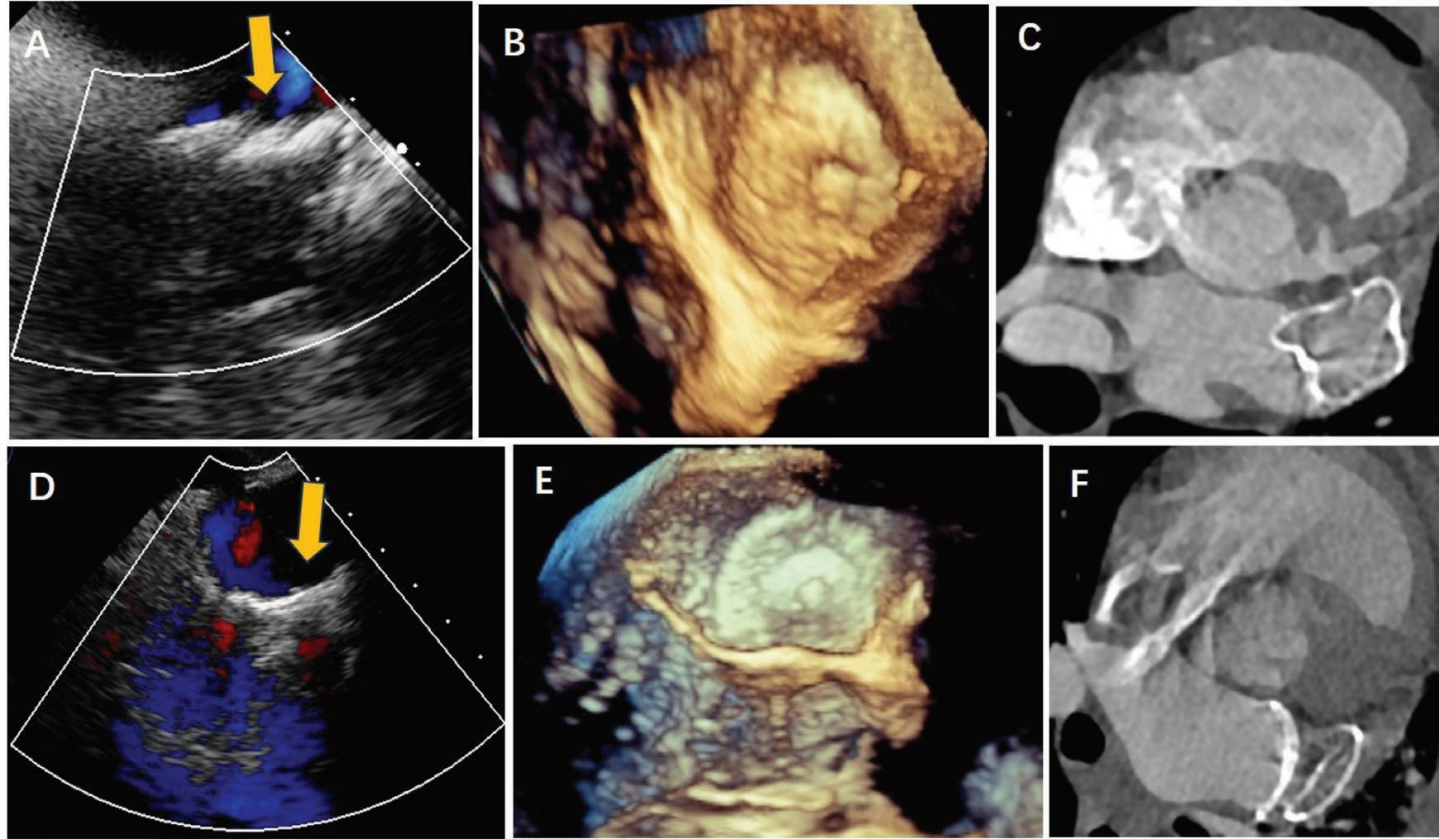
TEE and CT angiography images of the split occluder. After implantation in animal (A–C) TEE and computed tomography (CT) images at 90 days after implantation of the fixed disc. (A) The arrow points to the fixed disc. (B) Three‐dimensional view by TEE. (C) CT angiography image. All the images showed that the occluder was stable and had no obvious PDL and device‐related thrombosis. (D–F) TEE and CT images at 90 days after implantation of the combination of the fixed and covering discs. (A) The arrow showed the combination of the fixed and covering discs. (A) Three‐dimensional view by TEE. (C) CT angiography image. All the images showed that the occluder was stable and had no obvious PDL and device‐related thrombosis.

TEE and pulmonary vein CTV examination indicates that two of the dogs were monitored for a month, three for 3 months, and the remaining three for an additional 3 months prior to euthanasia. These examinations, conducted prior to euthanasia, confirmed that there was no evidence of thrombus on the surface of the occluder and also no detectable adverse effects on neighboring structures such as the mitral valve and pulmonary vein. In all cases, except one which underwent a shunt during the LAAO still exhibited a minor shunt, there was no noticeable residual shunt detected around the occluder (Figure 3). Data pertaining to the animal experiments are displayed in Table 1.

**Table 1:** Results of the animal experiments.

Characteristics	Animal (*n* = 8)
Male	5 (62.5%)
Weight, kg	30.2 ± 2.3
Type of LAA	
Antichicken wing shape	6 (75%)
Cauliflower shape	2 (25%)
Outer opening, inner diameter, mm	20.4 ± 1.2
Inner opening, inner diameter, mm	13.2 ± 1.1
Occluder type	
Single‐fixed disc	4 (50.0%)
Combination	4 (50.0%)

Device size	
22 mm (fixed disc)	2 (25.0%)
26 mm (fixed disc)	2 (25.0%)
20–28 mm (Combination)	2 (25.0%)
20–30 mm (Combination)	2 (25.0%)

Procedure	
Operation time, min	64.1 ± 10.5
Fluoroscopy time, min	22.3 ± 6.1
Success rate	8 (100%)

Follow‐up on 180 days	
Closure rate	7 (87.5%)
Residual shunt	1(12.5%)
Adverse events	0 (0%)


### 3.3. Gross Anatomy

**Figure 4:**
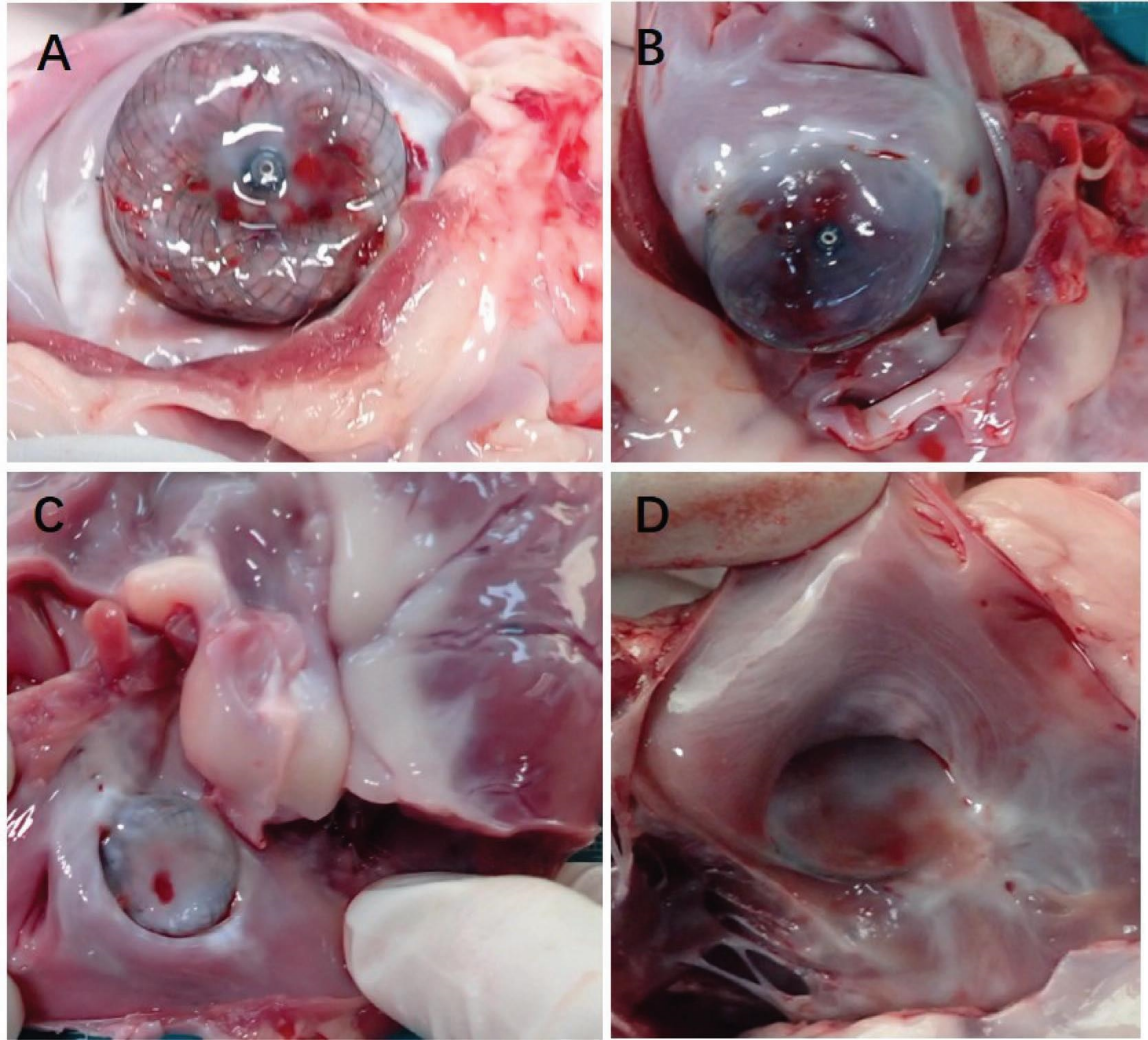
Gross observations of the occluder after implantation. (A) The occluder utilizing a single‐fixed disc occlusion began to endothelialize at the 1‐month follow‐up. (B) The occluder utilizing a combination of fixed and covering discs closure began to endothelialize at the 1‐month follow‐up. (C) At 3 months postimplantation, the occluder utilizing a single‐fixed disc occlusion was found to be completely endothelialized and without any device‐related thrombosis. (D) At 3 months postimplantation, the occluder utilizing a combination of fixed and covering discs was found to be completely endothelialized, without any device‐related thrombosis.

Two of the test dogs were euthanized 1 month following occluder implantation. The subsequent autopsies revealed that both the single fixed and the combination of the fixed and covering discs were in good positions. Furthermore, it was observed that the surface of the occluder had started to undergo endothelialization. Figure 4a,b depicts the gross observations made 30 days after the implantation of the split occluder. Three more test dogs were euthanized after 3 months, and the remaining three were euthanized 6 months after the implantation of the occluder. These autopsies revealed that the single‐fixed disc and the combined fixed and covering discs were securely positioned. It was further noted that the LAA had been completely sealed, and the occluder had undergone complete endothelialization and that the occluder had not induced any device‐related thrombosis. Additionally, the occluder had not negatively impacted the surrounding structure. Figure 4c,d displays the gross observations made 3 months following the implantation of the split occluder.

### 3.4. Short‐Term Results of First‐in‐Human Study

**Figure 5:**
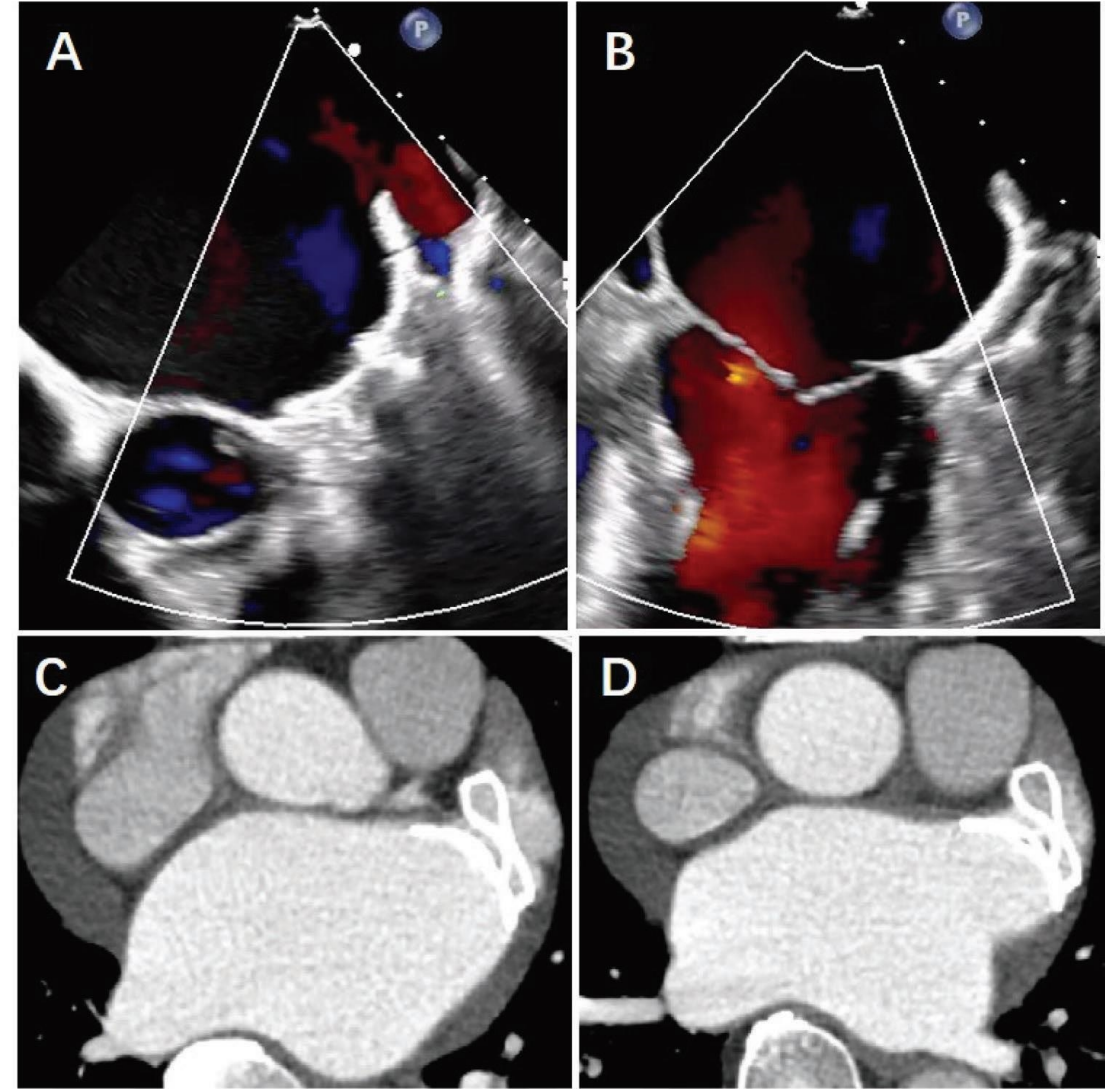
TEE and CT angiography images of the split occluder after implantation in human. (A–D) TEE and computed tomography (CT) images at 90 days after implantation of the combination of the fixed and covering discs. (A–B) TEE revealed that the occluder was stable and exhibited no evident PDL or device‐related thrombosis. (C–D) CT angiography revealed that the occluder was stable and exhibited no device‐related thrombosis but a minor PDL.

As shown in Table 2, the 12 individuals enrolled in the study consisted of seven males and five females, with an average age of 71.2 ± 4.5 years. The average CHA2DS2‐VASc score and HASBLED score were 5.1 ± 0.4 and 2.5 ± 0.8, respectively. Furthermore, 2 patients (16.7%) were diagnosed with paroxysmal AF, whereas 10 patients (83.3%) had persistent AF. In four instances, the LAA was occluded using a single‐fixed disc, whereas in the remaining eight cases, a combination of a fixed disc and a covering disc was utilized for a LAAO. Each procedure resulted in a complete occlusion, with no residual shunts exceeding 3 mm reported. Furthermore, all patients showed no PDL, except one exhibited a PDL of less than 1 mm. Notably, there were no documented major adverse events, operation‐related complications, or device‐related serious adverse events within 3 months postoperation in any of the subjects. The procedure and follow‐up findings are detailed in Table 3. In addition, TEE revealed that the occluders exhibited excellent morphology, with no thrombus on the occluder′s surface, and no adverse effects on neighboring structures such as the mitral valve and pulmonary vein. No noticeable residual shunt was detected around the occluder in the majority of cases, except for a minor shunt observed in one case. Five patients received pulmonary vein CTV scanning 90 days after the implantation of the occluder, which evidenced neither thrombus on the occluder′s surface nor PDL. Figure 5 shows the TEE and pulmonary vein CTV images of one patient at 90 days postprocedure.

**Table 2:** Baseline characteristics of first in‐human study.

Characteristics	Patients (*n* = 12)
Male, %	7 (58.3%)
Age, years	71.2 ± 4.5
BMI, kg/m^2^	22.3 ± 1.7
Type of atrial fibrillation	
Paroxysmal AF	2 (16.7%)
Persistent AF	10 (83.3%)
CHA2DS2‐VASc score	5.1 ± 0.4
HASBLED score	2.5 ± 0.8
NYHA level	1.5 ± 0.3
Congestive heart failure	1 (8.3%)
Hypertension	4 (33.3%)
Diabetes mellitus	3 (25.0%)
Stroke	2(16.7%)
Coronary heart disease	2 (16.7%)
Valvular disease	0 (0.0%)

**Table 3:** Acute results of procedure and 90‐day follow‐up of human study (*N* = 12).

Characteristics	Patients (*n* = 12)
Success rate	
Postoperation technical success rate	12 (100.0%)
LAAO success rate	12 (100.0%)
Occluder type	
Single‐fixed disc	4 (33.3%)
Combination	8 (66.7%)
Procedural time	
Mean procedural time, min	43.6 ± 6.8
Fluoroscopy time, min	12.4 ± 1.6

Major adverse events(90 days)	
All cause of death	0 (0.0%)
Stroke (including ischemic stroke, hemorrhagic stroke and transient ischemic attack)	0 (0.0%)
Systemic thromboembolism	0 (0.0%)
Device migration	0 (0.0%)
Device related thrombosis	0 (0.0%)
Pericardial tamponade	0 (0.0%)
Infection	0 (0.0%)

Peridevice leak (PDL)	
None	10 (83.4%)
≤ 1 mm	1 (8.3%)
1 < PDL ≤3 mm	1 (8.3%)
≥ 3 mm	0 (0.0%)


## 4. Discussion

This study evaluated the efficacy and safety of a split LAA occluder in both animal and human subjects, assessing acute procedural outcomes followed by months of review. Follow‐up TEE and pulmonary vein CTV revealed no noticeable PDL and documented no adverse events, including device detachment or cardiac perforation. Complete device endothelialization was observed at the 90th and 180th days postprocedure, indicating successful integration. Moreover, the first‐in‐human study yielded highly successful outcomes, with no adverse events reported in any of the 12 cases.

AF is a particularly prevalent type of cardiac arrhythmia. It is estimated that between 10% and 40% of AF patients require hospitalization annually [2]. AF elevates the risk of ischemic stroke threefold to fivefold, and the mortality rate of AF‐related strokes is twice that of other strokes [9]. Current guidelines state that the utilization of oral anticoagulants (OAC) for thromboembolism prevention in patients with AF is a Class IA recommendation. Direct or novel OACs are commonly employed to prevent AF‐related strokes [10]. Nevertheless, a subset of patients with AF and at a high risk of stroke are not well‐suited for long‐term OAC anticoagulation owing to their heightened risk of bleeding. LAAO, which has an effectiveness comparable to that of OAC [3, 11–13], is increasingly recommended for patients with AF that should not be prescribed OACs. Outside of China, the most popular occluders are currently produced by Watchman and Amulet; within China, occluders such as LAmbre and LACbes have been approved for clinical trials. However, these devices may not be the right size for the diverse range of tissue structures found within the LAA of different patients, leading to either inadequate or partial occlusion. There is, thus, an urgent need for a novel LAA occluder. This device should be adaptable to the size of the occlusion required based on the morphology of the LAA as discovered midprocedure. Such a device could significantly enhance the adaptability of the LAA occluder to the range of shapes that different LAAs present, providing a more effective and tailored solution for patients suffering from AF.

The split LAA occluder used in this study was independently researched and developed in China. The occluder comprises a fixed disc and a covering disc, with the former being separated from the latter. The fixed disc can be employed in isolation as a plug‐type occluder, or it can be connected to the covering disc, serving as a cover‐type double‐disc occluder. The plug‐type occluder, when deployed as a standalone device, lacks an external coverage component. The size specifications of the fixed umbrella and the covering disc can be adjusted as required. The split LAA occluder overcomes the problems faced by existing products and possesses the advantages of both the inner plug and the outer cover types of occluder, further improving occluder flexibility and widening the scope of its application. This is demonstrated by the fact that no significant PDL was observed in each of the 12 human tests and the 8 canine tests. It should be noted that the fixed disc and the covering disc could not be unlocked again to avoid connection loosening. In case of improper combination, it is only possible to choose a new occlude combination.

In animal research, performing the atrial septal puncture may be challenging owing to variations in DSA projection angles. Cardiac tamponade has been documented in animal experiments during atrial septal puncture procedures [14]. However, in our study, utilizing ICE guidance, eight test animals successfully underwent atrial septal puncture and the implantation of a LAA occluder, indicating the safety and effectiveness of ICE‐guided procedures. All animals in this group were successfully implanted with a split occluder without any perioperative complications such as cardiac tamponade or death. The postoperation left atrial angiographies revealed a small shunt in one dog, whereas the remaining seven exhibited complete occlusion, an 87% occlusion rate. Previous studies have linked residual shunts exceeding 5 mm in the LAA to a risk of thrombosis [15], and so ensuring the complete occlusion of the LAA is crucial for the effectiveness of the procedure. Although one animal in this study experienced a minor residual shunt postoperation, no thromboembolic events were observed during follow‐up, suggesting that a small residual shunt was within tolerance limits. Contrasting with many previous studies [16, 17] that utilized TEE to evaluate LAA occluders, this animal experiment employed a multifaceted approach, including TEE, pulmonary vein CTV, and gross anatomical dissection, to comprehensively assess the safety and efficacy of both types of split occluder. The results of this experiment confirm that the occluder begins endothelialization within 1 month, with significant thickening and complete endothelialization at 3 months, without affecting surrounding biological structures. This comprehensive evaluation underscores the reliability and efficacy of the employed methods and devices in this study.

### 4.1. Study Limitations

Several limitations should be acknowledged. First, the study′s sample size of eight dogs with a 6‐month follow‐up period is relatively small and limits the scope of the findings. Therefore, large‐scale studies with extended follow‐up durations are essential to validate these results. Secondly, the test dogs were all healthy, meaning that their atria size differs from that of dogs suffering from AF. This suggests that further clinical trials are needed to fully evaluate the device′s effectiveness and safety in a broader range of scenarios and patient populations.

## 5. Conclusions

The animal experiment and first in‐human study demonstrated the adaptability of the split occluder to various LAA morphologies, along with its capacity for rapid endothelialization. This suggests that it could be a viable device‐based alternative for LAAO treatment, particularly for patients with complex LAA morphology.

## Author Contributions

Mingfei Li and Dawei Lin contributed equally to this work.

## Funding

This study was supported by Shanghai Clinical Research Center for Interventional Medicine (19MC1910300).

## Disclosure

All authors have reported that they have no relationships relevant to the contents of this paper to disclose.

## Conflicts of Interest

The authors declare no conflicts of interest.

## Data Availability

The data that support the findings of this study are available on request from the corresponding authors. The data are not publicly available due to privacy or ethical restrictions.
